# The age factor in Alzheimer’s disease

**DOI:** 10.1186/s13073-015-0232-5

**Published:** 2015-10-20

**Authors:** Rita Guerreiro, Jose Bras

**Affiliations:** Department of Molecular Neuroscience, Institute of Neurology, University College London, 1 Wakefield Street, London, WC1N 1PJ UK

## Abstract

Alzheimer’s disease is the most common type of dementia, and it is characterized by a decline in memory or other thinking skills. The greatest risk factor for Alzheimer’s disease is advanced age. A recent genome-wide study identified a locus on chromosome 17 associated with the age at onset, and a specific variant in *CCL11* is probably responsible for the association. The association of a protective haplotype with a 10-year delay in the onset of Alzheimer’s disease and the identification of a *CCL11* variant with possible functional roles in this association might allow the future development of immunomodulators with the potential to halve disease incidence.

Alzheimer’s disease (AD) is a common and multifactorial neurodegenerative disease. It is one of the leading causes of death around the world and is associated with substantial direct and indirect costs for families and societies. The vast majority of AD cases have a late onset (usually after 65 years of age); the disease is rare among younger individuals. Incidence of AD is known to increase with age, and age is the most important risk factor for the development of AD. The ability to delay the age at onset (AAO) of this disease through preventive or therapeutic approaches would have significant benefits, but no therapies have been successful in achieving this important goal [1, 2]. A recent study by Lalli et al*.* [3] used genome-wide technologies to identify genetic modifiers of AAO in a unique cohort comprising an extended Colombian family carrying the *PSEN1* p.E280A mutation, and the outcome might be useful for guiding the search for therapies.

## Age at onset as a highly heritable factor in AD

AAO is highly heritable in AD families. In early-onset cases, mutations in three genes are known to account for around half of familial cases [4]: Amyloid Precursor Protein (*APP*), Presenilin-1 (*PSEN1*) and Presenilin-2 (*PSEN2*). However, the genes involved in AAO variance mostly remain to be identified; linkage and candidate gene studies have established several possible associations with different loci, but the only consistently replicated modulator of AD AAO, in both familial and sporadic cases, is also the strongest genetic risk locus for the development of the disease (*APOE*) [5]. More recently, Naj et al*.* [6] performed a large genome-wide association study (GWAS) in over 9000 patients to detect effects of known AD risk loci in modifying AAO. They confirmed the association of *APOE* Ɛ4 allele with earlier onset and identified associations with *CR1*, *BIN1* and *PICALM*. Burden analyses showed that *APOE* contributed 3.7 % of AAO variation; the other nine loci studied contributed 2.2 % when considered together [6].

Many of these studies suffered from confounders, such as genetic and phenotypic heterogeneity, population stratification or inaccurate phenotyping. In fact, AAO of a disease is often a very difficult parameter to record with precision when assessed retrospectively. Lalli et al*.* [3] reduced the effects of many of these confounders by assessing a unique cohort of 72 Colombian patients carrying the same *PSEN1* mutation (p.E280A) who are part of an extended family and who have been subjected to careful prospective phenotyping. By analyzing the genome sequence of individuals with different AAOs, the authors [3] identified a locus on chromosome 17q12 that showed a significant association with AAO in this cohort. Within this associated locus, they were able to identify a rare variant (p.A23T) in *CCL11* (which encodes the chemokine eotaxin-1) associated with a 10-year delay in AAO of AD [3].

## Age and immune response in AD

What seemed like a farfetched idea a few years ago is now a well established fact in AD: inflammatory and immune responses have a significant role in its development and progression. Several of the genetic loci associated with AD risk contain genes with known roles in inflammation, the complement system and the immune response in general (*ABCA7*, *CLU*, *CR1*, *MS4A4E*/*MS4A6A*, *CD33*, *EPHA1*, *HLA-DRB5*, *HLA-DRB1*, *INPP5D*, *MEF2C* and *TREM2*). Pathway analyses of GWAS data have identified the immune response as important in AD, and an integrated network analysis of genome and transcriptome data identified the immune and microglia module as significant for AD and *TYROBP* as the driver gene for this module [7, 8].

Microglial activation and monocyte/macrophage-mediated inflammatory responses are currently particularly interesting areas of research on AD. To evaluate the relationship between known AD risk loci, Chan et al. [9] recently conducted a protein quantitative trait analysis in monocytes and showed that the *NME8* risk allele influences protein tyrosine kinase 2 β (PTK2B), the *CD33* risk allele influences triggering receptor expressed on myeloid cells 2 (TREM2) and the *TREM1* risk allele is associated with a decreased TREM1/TREM2 ratio. Interestingly, the authors [9] also uncovered potential differences associated with age in the expression of genes in the *TREM* locus. *TREM1* expression was found to increase with advancing age in younger but not in older individuals, and *TREM1* variants were found to affect *TREM2* expression in younger but not older people [9].

## AAO as a therapeutic target

The ability to delay the AAO of AD through preventive or therapeutic approaches would have significant benefits. A 2012 study found a protective variant in *APP*, which suggests that manipulating the amyloid pathway could be a successful approach to reducing AD [10]. One can predict that other elements participating directly or indirectly in the proteolytic processing of APP will also be good therapeutic targets to modulate the disease.

Similarly, the variant found in *CCL11* in the most recent study by Lalli et al. [3] also has a high potential as a therapeutic target. It encodes eotaxin, and the concentration of eotaxin has been shown previously to rise with age both in people and in mice. Eotaxin has also been identified as potentially one of the most deleterious factors for cognitive function and neurogenesis in aging by parabiosis studies in which the blood of two mice, one young and one old, was shared through the surgical fusion of vascular systems [11]. The variant identified by Lalli et al*.* [3] (p.A23T) is located in the protein signal peptide cleavage site, suggesting a functional role for the variant, possibly through the binding with its receptor (CCR3). The enhanced secretion of eotaxin by cells transfected with this variant [3] validate the functional significance of the variant, but, given the protective role of lower levels of eotaxin, the finding of increased cell secretion is in the opposite direction to what was expected. These findings clearly demonstrate the need to better understand the biology of genomes and emphasize the need to further characterize the molecular biology of these genomic regions.

## Concluding remarks

It is important to understand how the recent findings from isolated and endogamous cohorts will translate to other populations, and what the implications are for the understanding of late-onset AD. Although single rare variants will not have high population-attributable risks, they will be extremely important for individual risk prediction and for the understanding of biology and pathobiology. Likewise it will be important to understand how findings made in early-onset familial cases will translate to the most common and sporadic late-onset AD. Two observations support significant commonalities between these two forms of disease, suggesting that factors important for early-onset and familial cases will also be important for late-onset and sporadic AD: first, the *APOE* Ɛ4 allele not only modulates the AAO of AD in a sporadic setting, but also in carriers of presenilin mutations, and second, the identification of the protective effect of *APP* p.A673T in the Icelandic population showed that amyloid β has crucial roles in both early- and late-onset AD [10].

The mechanisms through which eotaxin and other peripheral immune molecules contribute to the disease process are still to be determined, but the identification of an association between *CCL11* and AAO in the studied Colombian kindred [3], if validated, can potentially be translated into immunomodulating therapies able to delay the onset of AD in the future.

## Abbreviations

AAO: Age at onset
AD: Alzheimer’s disease
GWAS: Genome-wide association study
